# Effects of age, HIV, and HIV-associated clinical factors on neuropsychological functioning and brain regional volume in HIV+ patients on effective treatment

**DOI:** 10.1007/s13365-018-0679-4

**Published:** 2018-10-08

**Authors:** Natalia Gawron, M. Choiński, B. Szymańska-Kotwica, A. Pluta, M. Sobańska, A. R. Egbert, A. Desowska, T. Wolak, A. Horban, E. Firląg-Burkacka, P. Bieńkowski, H. Sienkiewicz-Jarosz, A. Scińska-Bieńkowska, B. Biswal, S. M. Rao, R. Bornstein, E. Łojek

**Affiliations:** 1 The Faculty of Psychology, University of Warsaw, Stawki 5/7, 00-183 Warsaw, Poland; 2 Nencki Institute of Experimental Biology, Polish Academy of Sciences, 3 Pasteur St., 02-093 Warsaw, Poland; 3 Hospital for Infectious Diseases, 37 Wolska St., 01-201 Warsaw, Poland; 4 Institute of Physiology and Pathology of Hearing, 17 Mokra st, Kajetany, 05-830 Nadarzyn, Poland; 5 The Department of Biomedical Engineering, New Jersey Institute of Technology, University Heights, Newark, NJ 07102 USA; 6 Institute of Psychiatry and Neurology, 9 Sobieskiego St., 02-957 Warsaw, Poland; 7 Department of Neurology I, Institute of Psychiatry and Neurology, 9 Sobieskiego St., 02-957 Warsaw, Poland; 8 Department of Pharmacology, Institute of Psychiatry and Neurology, 9 Sobieskiego St., 02-957 Warsaw, Poland; 9 The Cleveland Clinic, 9500 Euclid Ave., Cleveland, OH 44195 USA; 10 The College of Medicine, The Ohio State University, 370 W. 9th Ave., Columbus, OH 43210 USA

**Keywords:** HIV, Aging, Neuropsychological functioning, Brain volume, cART, CD4 cell count nadir

## Abstract

It is yet unclear if people infected with human immunodeficiency virus (HIV+) on stable, combined antiretroviral therapies (cARTs) decline with age at the same or greater rate than healthy people. In this study, we examined independent and interactive effects of HIV, age, and HIV-related clinical parameters on neuropsychological functioning and brain regional volume in a sizable group of Polish HIV+ men receiving cART. We also estimated the impact of nadir CD4 cell count, CD4 cell count during participation in the study, duration of HIV infection, or duration of cART along with age. Ninety-one HIV+ and 95 control (HIV−) volunteers ages 23–75 completed a battery of neuropsychological tests, and 54 HIV+ and 62 HIV− of these volunteers participated in a brain imaging assessment. Regional brain volume in the cortical and subcortical regions was measured using voxel-based morphometry. We have found that HIV and older age were independently related to lower attention, working memory, nonverbal fluency, and visuomotor dexterity. Older age but not HIV was associated with less volume in several cortical and subcortical brain regions. In the oldest HIV+ participants, age had a moderating effect on the relationship between the duration of cART and visuomotor performance, such as that older age decreased speed of visuomotor performance along with every year on cART. Such results may reflect the efficacy of cART in preventing HIV-associated brain damage. They also highlight the importance of monitoring neuropsychological functioning and brain structure in HIV+ patients. This is particularly important in older patients with long adherence to cART.

**Electronic supplementary material:**

The online version of this article (10.1007/s13365-018-0679-4) contains supplementary material, which is available to authorized users.

## Introduction

Due to combination antiretroviral therapy (cART), human immunodeficiency virus (HIV) infection has become a manageable, chronic illness and many HIV+ patients reach advanced age. In Europe, the proportion of individuals presenting several years after being infected is high among people older than age 50 (ECDC/WHO 2017). Also, a significant proportion of new diagnoses are being made in this age group (Tavoschi et al. 2017). Studies suggest that HIV may continue to affect the brain even in the presence of cART. The impact of the infection on the aging brain structure and cognitive functioning is not yet fully understood. In this article, we depict the research evidence showing the influence of HIV infection, age, and HIV-associated clinical factors on neuropsychological performance and brain volume in HIV-positive (HIV+) Polish men receiving cART.

The introduction of cART has decreased the incidence of HIV-associated neurological complications and dementia. Neuropsychological abnormalities, however, remain common despite improved viral suppression (Heaton et al. 2010). Even HIV+ patients with undetectable HIV-1 RNA concentrations in the plasma show declines in attention, memory, psychomotor ability, or executive functions (Cysique et al. 2014; Muñoz-Moreno et al. 2008; Saktor et al. 2010). As individuals with HIV have longer life expectancies, the prevalence of cognitive impairment is likely to increase in the elderly (Sheppard et al. 2015). It is unclear, however, if HIV+ patients decline with age at the same or greater rate than matched HIV− comparators, i.e., if the HIV-age effects on cognition are independent or interactive. Many studies have demonstrated that the degree to which cognitive outcomes decline due to HIV is not increased by older age (Seider et al. 2016; Valcour et al. 2011). Yet, significant interactive effects of HIV and age have been reported in one longitudinal study in which older HIV+ individuals declined in verbal memory over one year at greater rate than demographically similar HIV− controls (Seider et al. 2014).

There also is substantial evidence that even in effectively treated HIV+ individuals, the infection contributes to decreases in regional brain volume. Cortical reductions due to HIV have been found in frontal, orbitofrontal, temporal, cingulate, primary motor, and sensory areas (Küper et al. 2011; Sandford et al. 2017; Towgood et al. 2012). Subcortical reductions due to HIV have been revealed within the amygdala, caudate, corpus callosum (Ances et al. 2012; Guha et al. 2016), basal ganglia (Küper et al. 2011), brainstem and thalamus (Sanford et al. 2017), or white matter (Hua et al. 2013). It is not well understood if HIV infection accelerates brain changes associated with aging. To date, Pfefferbaum and collaborators (Pfefferbaum et al. 2014) reported cortical volume decreases intensifying with age in older HIV+ individuals. Cardenas and collaborators (Cardenas et al. 2009) noted faster white matter volume loss in HIV+ patients with viral suppression than in matched HIV− controls, while Seider and collaborators (Seider et al. 2016) elucidated more pronounced increases in white matter hyperintensities in older HIV+ patients than in controls.

The development of brain abnormalities and cognitive deficits in HIV+ patients may be related to parameters such as the lowest CD4 lymphocyte cell count in blood (nadir CD4), detectable plasma HIV RNA level, or duration of cART (Ellis et al. 2011; van den Dries et al. 2017). CD4 cells are a type of lymphocyte cells. They stimulate other immune cells to fight infection. Nadir CD4 cell count is the lowest point to which CD4 count has dropped due to HIV infection, i.e., the greatest immunosuppression, and is a particularly significant predictor of neurological complications and cognitive impairment (Valcour et al. 2006). Strong evidence demonstrates that the incidence of neuropsychological deficits is highest in HIV+ patients who experienced CD4 cell counts below 200 cells/mm^3^ (Ellis et al. 2011; Muñoz-Moreno et al. 2008; Seider et al. 2014). CD4 recovery with continuous viral suppression also takes longer in patients who initiate cART at low CD4 cell counts (Costagliola et al. 2014).

Neuroimaging studies of HIV+ populations have reported significant associations between low nadir CD4 cell count and brain regional volume. Nadir CD4 levels have been correlated with volume reductions in gray matter (Küper et al. 2011; McCombe et al. 2013), white matter, and subcortical gray matter (Hua et al. 2013), as well as increased amounts of the cerebrospinal fluid (Jerningan et al. 2011; Su et al. 2016). Reductions in brainstem white matter, internal capsule, globus pallidus, and caudate also have been reported (Sanford et al. 2017). Low Current CD4 cell count and detectable plasma HIV RNA levels have been reported as correlates of ventricular enlargement and reductions in the basal ganglia (Hua et al. 2013).

It is not clear if the extent of cognitive decline and neural injury may be determined by interactions between clinical factors and older age. It has been hypothesized that older HIV+ patients are at risk of accelerated aging of the immunological system (Hong Banks 2015). Older age also may constraint the capacity of treated patients to reconstitute their CD4 cell resources (Appay et al. 2011).

Despite these findings, our knowledge on aging in HIV+ populations remains limited. The evaluation of HIV-age interactions within a sizable group of relatively healthy aviremic HIV+ subjects on cART would add to our scientific understanding of this disease. The current cross-sectional study investigated independent and interactive effects of HIV infection and aging on neuropsychological functioning and gray and white matter as measured with brain morphometry in HIV-positive Polish men with viral suppression below the level of 60 copies/mL. Our second objective was to explore the impact of HIV-associated clinical parameters and duration of infection while controlling for age. In accordance with the existing evidence, we hypothesized that both HIV infection and age would have adverse effects on cognitive status and brain regional volume despite stable antiretroviral regimen and undetectable viral loads in plasma.

## Method

### Participants

HIV+ participants were selected through structured clinical interview among the patients at the Hospital of Infectious Diseases in Warsaw. Inclusion criteria for the study were as follows: male, age greater than 25 years, HIV infected through sexual contact, HIV positive on the ELISA and Western blot tests, and being successfully treated with cART for at least ten months before the assessment (viral load in blood serum < 60 copies/μL). Exclusion criteria were active opportunistic diseases (tuberculosis, mycobacterium avium complex, fungal infections, toxoplasmosis, herpes, cytomegalovirus, bacterial pneumonia, and sepsis salmonella), active co-infection with hepatitis C virus (HCV), active syphilis or neurosyphilis, head trauma with loss of consciousness greater than 30 min, developmental disorders, dementia, severe psychiatric conditions, liver or renal insufficiency, less than 12 years of education, illicit drugs use, and alcohol abuse.

Clinical HIV-related parameters were collected from the medical history and standard blood laboratory testing. They included CD4 cell count during participation in the study, nadir CD4 cell count, current plasma viral load, highest plasma viral load, time since HIV diagnosis in years, and time since initiation of the first cART regimen in years. We collected data on previous medical conditions that potentially could affect cognitive performance, including psychiatric disorders, hypertension, diabetes, depression, anxiety, and alcohol consumption. HIV-negative (HIV−) control subjects similar in age and education were selected from the local healthy population, and all underwent the same inclusion/exclusion criteria assessment, and neuropsychological and imaging protocols as the HIV+ subjects. Controls underwent blood testing to exclude HIV or HCV infection. Twelve subjects were excluded due to learning disorders and overall low cognitive performance, plasma viral load during the study > 60 copies/mL, gross brain structural abnormalities, or HIV detection.

A sample of 91 HIV+ and 95 HIV− participants were retained for the analysis of the effects of HIV, age, and clinical factors on neuropsychological performance. Of those, 54 HIV+ and 62 HIV− controls participated in brain imaging assessment (MRI subsamples). We implemented analyses of neuropsychological data on the entire study population, expecting more reliable results from a larger population and being highly motivated to show the results of all participants. Most of the HIV+ participants were cognitively normal according to HIV-Associated Neurocognitive Disorder (HAND) criteria (81.5% were classified as no HAND, Egbert et al. 2018).

The study was carried out according to the Code of Ethics of the World Medical Association (Declaration of Helsinki). All participants provided written informed consent. The Ethics Committee of the University of Warsaw approved the consent, recruitment procedure, and course of the examination.

### Neuropsychological assessment

All participants completed a battery of neuropsychological tests administered by one of six certified neuropsychologists. The battery covered multiple domains that often are impaired in HIV infection (Becker et al. 2015). The Digit Span forward and backward subtest of the Polish adaptation of the Wechsler Adult Intelligence Scale–Revised (WAIS–R [PL]; Brzeziński et al. 2004), the Corsi Block Tapping Test forward and backward (Corsi 1972), and the Colour Trails Test (CTT) parts 1 and 2 (Łojek Stańczak 2012) were used to measure attention and working memory. The Wisconsin Card Sorting Test (WCST; Jaworowska 2002) and the Ruff Figural Fluency Test (RFFT; Łojek Stańczak 2005) were used to assess executive functions, and the Grooved Pegboard Test place and remove task (Haaland et al. 1977) measured visuomotor dexterity. The California Verbal Learning Test (CVLT; Łojek Stańczak 2010) was used to estimate learning and memory after short and 20-min long delays, while the Verbal Fluency subtest was used to measure letter and category verbal fluency (Szepietowska Gawda 2011). The Vocabulary test of the WAIS–R (PL) (Brzeziński et al. 2004) assessed language and premorbid intelligence. The Mini-Mental State Examination (MMSE; Stańczak 2010) was administered before the neuropsychological examination to eliminate participants with dementia.

### Brain morphometry

To study brain regional volumes, structural magnetic resonance images of subjects were acquired using a 3T Siemens TIM TRIO whole-body magnetic resonance scanner with 12-channel head coil. The T1-weighted images were acquired with the following acquisition parameters: TE = 2.21 ms, TR = 1900 ms, TI = 900 ms, flip angle = 9°, field of view = 260 mm × 288 mm, slice thickness = 0.9 mm, number of slices = 208, image matrix = 290 × 320 what gives isotropic voxel size 0.9 × 0.9 × 0.9 mm, pixel bandwidth = 200 Hz/pix, iPAT = 2, and TA = 5 min.

Initially, images were visually inspected by a biomedical engineer and a radiologist for artifacts and/or structural abnormalities unrelated to HIV (e.g., tumors). FreeSurfer (http://surfer.nmr.mgh.harvard.edu/) (Fischl 2012) was used to derive morphometric measurements of individual brain regions. The analysis was carried out with standard surface-based and volume-based streams including the following: (a) for surface-based: volume registration with the MNI305, intensity normalization, the skull stripping, segmentation, tessellation of the gray matter white matter boundary, automated topology correction, and surface deformation following intensity gradients; (b) for volume-based: volume registration with the MNI305, initial volumetric labeling, intensity normalization, and a high dimensional nonlinear volumetric alignment to the MNI305, after the preprocessing the volume was labeled again. Image quality was assessed by visual inspection; skull stripping and segmentation results met quality assurance standards for both cortical and subcortical segmentation.

The regions distinguished analyzed in the present study were as follows: left and right hemisphere cortical gray matter, total cortical gray matter, left and right hemisphere cortical white matter, total cortical white matter, total gray matter, subcortical gray matter; bilaterally: cerebellum white matter, cerebellum cortex, thalamus, caudate, putamen, pallidum, hippocampus, amygdala, accumbens, vessels, choroid plexus, lateral ventricle, then brainstem, 3rd ventricle, 4th ventricle, 5th ventricle, white matter hypointensities, optic chiasm; and finally posterior, midposterior, central, and anterior cingulate. To adjust for the differences in head size, measurements for individual brain regions were divided by the intracranial cavity volume. The impact of HIV and age on cortical regions was analyzed in a separate study (Pluta et al. in press).

### Statistical methods

Group differences in demographics and laboratory measures were examined using *t* test for continuous variables and Pearson’s chi-square tests for categorical variables. Outliers greater than 3 standard deviations (*SD*s) from *z*-mean were winsorized (1.4% of 5022 cases in 27 neuropsychological and 4 clinical variables). Preliminary analyses revealed significant correlations between WAIS–R (PL) Vocabulary, a measure of language and premorbid verbal intelligence, and all neuropsychological tests in in HIV+ and HIV− groups. According to studies, premorbid intelligence may influence changes in cognitive functioning in HIV+ patients (Basso Bornstein 2000; Heaton et al. 2015). We then decided to control for the WAIS–R (PL) Vocabulary when estimating the effects of HIV, age, or clinical variables on neuropsychological performance. The WAIS–R (PL) Vocabulary variable also was included when estimating the effects of HIV, age, or clinical indices on brain regional volume, as in previous studies in which demographic or education variables were entered to account for variance in brain volumes (Sanford et al. 2017).

Independent effects of HIV and age on neuropsychological measures and brain regional volumes were investigated in a series of multivariate linear regression models. In every regression model, HIV infection as a dichotomous factor and age as a continuous factor were included to account for variance in neuropsychological or morphometry measures; WAIS–R (PL) Vocabulary was also entered as continuous predictor variable. Interactive effects of HIV and age on cognition and brain volume were examined with a separate series of regression models in PROCESS version 3 (Hayes 2013). Here, the aim was to determine if aging moderated the extent to which neuropsychological outcomes or brain volume depended on HIV status. In each regression model, HIV status was the independent dichotomous variable, age was the continuous moderator variable (W), and WAIS–R (PL) Vocabulary score was the covariate. Model number 1 with default 5000 Bootstrap Samples for indirect effects, 95% confidence interval, mean centering for products, and none heteroscedasticity-consistent inference was used.

Effects of age and HIV-related parameters—nadir CD4 cell count, current CD4 cell count, highest viral load, duration of HIV infection in years, duration of cART in years—on cognition and brain volume were also estimated with multivariate linear regression models. The interactive effects of these predictors were examined with a separate series of regression models in PROCESS. In every model, clinical parameters were entered as the independent variable, age was the continuous moderator variable (W), and WAIS–R (PL) Vocabulary score was the covariate. Model settings were identical as in foregoing analyses. Prior to analyses, highest viral load, duration of infection, and years on cART were log transformed to normalize the distributions. Highest viral load was not included in further analyses because its log-transformed distribution departed from normality.

All statistical analyses were performed using IBM SPSS Statistics version 24. Effects with *R*^2^ < .1, *R*^2^-change < .1, or *R*^2^-change due to interaction < .1 are not reported in this article.

## Results

### Socio-demographic and clinical characteristics

There were no significant differences in age, years of formal education, and MMSE between HIV+ and HIV− groups or between MRI HIV+ and HIV− subgroups. The ratio of homosexual to heterosexual participants was higher in the HIV+ group than in the HIV− group (*χ*^2^ (4, *n* = 186) = 31.062, *p* < .001) as well as in the MRI HIV+ group in comparison to the MRI HIV− group (*χ*^2^ (4, *n* = 116) = 19.285, *p* < .002). This was due to the small proportion of homosexual men in the local community (Table 1). Age was normally distributed in all groups.

**Table 1:** Characteristics of the study sample

	HIV+ *N* = 91 *M* (*SD*)	HIV− *N* = 95 *M* (*SD*)	*t/χ* ^*2*^	MRI HIV + *N* = 54 *M* (*SD*)	MRI HIV − *N* = 62 *M* (*SD*)	*t/χ* ^*2*^
Age (years)	41.2 (11.9)	44.3 (12.5)	NS	41.1 (12.1)	43.8 (12.5)	NS
Education (years)	16.4 (2.7)	16.4 (2.7)	NS	16.1 (2.7)	16.6 (2.9)	NS
Employed full time, part time, or mandate job (% participants)	78 (72)	79 (84)	NS	42 (79.2)	53 (85.5)	NS
Only homosexual behaviors (% participants)*	74 (81.3)	46 (48.4)	< .001	44 (81.5)	33 (53.2)	< .002
MMSE	29.1 (1.1)	29.3 (1)	NS	29 (1.1)	29.3 (.9)	NS
CD4 cell count (cells/μL)	597.3 (213.2)	NA	NA	589.1 (182.8)	NA	NA
Nadir CD4 cell count (cells/μL)	272.9 (145.4)	NA	NA	270.6 (130.7)	NA	NA
Plasma viral load (copies/mL)	38.3 (8.6)	NA	NA	37.9 (9.9)	NA	NA
Highest plasma viral load (copies/mL)	204,158.1 (440, 934.6)	NA	NA	175,800.8 (463, 804.3)	NA	NA
Years since HIV diagnosis	5 (4.8)	NA	NA	5 (5)	NA	NA
Years on cART	5 (4.9)	NA	NA	5.1 (5.1)	NA	NA
Past co-infection HIV/HCV (% participants)	11 (12.2)	NA	NA	8 (14.8)	NA	NA
Past syphilis or neurosyphilis (% participants)	47 (52.8)	0	NA	27 (50)	0	NA

*NS* not significant, *NA* not applicable, *SD* standard deviation

*Sexual behaviors were classified as only homosexual, mainly homosexual sometimes heterosexual, equally homosexual and heterosexual, mainly heterosexual sometimes homosexual, or only heterosexual

### Effects of HIV and age on neuropsychological performance

Independent effects of HIV infection and aging on neuropsychological functioning were estimated, including the results of all participants. To control for the familywise error rate, Bonferroni correction was applied setting the *p* value to be *p* < 0.05/total number of variables tested, i.e., 2 predictors (HIV, age) + 1 covariate (Vocabulary) + 26 dependent variables = 0.002 for each predictor effect. Neuropsychological characteristics of the studied groups are in the Appendix.

Regression coefficients shown in Table 2 demonstrate that HIV and age explained significant variability in Corsi Block Tapping backward, Digit Span backward, number of unique designs drawn in RFFT, and time of removing pegs in GPT (all effects *p* < 0.002 except for the effect of age for Corsi Block Tapping forward *p* = .005). Both HIV and older age were related to lower test performance.

**Table 2:** Significant effects of HIV and age on neuropsychological performance in the entire HIV+ and HIV− groups

Neuropsychological measure	HIV		Age			
	*β*	*p*	*β*	*p*	Adjusted *R*^2^	*p*
Attention/working memory						
Corsi Block Tapping forward	− .168	< .02	− .265	< .001	.137	< .001
Corsi Block Tapping backward	− .287	< .001	− .238	< .001	.207	< .001
WAIS–R (PL) Digit Span backward	− .239	< .001	− .192	= .005	.184	< .001
CCT 1 Time	.042	= .557	.370	< .001	.148	< .001
CTT2 time	.069	= .310	*.*435	< .001	.222	< .001
Executive						
RFFT unique designs	− .242	< .001	− .289	< .001	.210	< .001
WCST percent errors	− .010	= .878	.273	< .001	.231	< .001
WCST percent conceptual responses	− .003	= .969	− .253	< .001	.186	< .001
Learning						
CVLT list A Trials 1–5	.052	= .427	− .431	< .001	.268	< .001
CVLT list B	− .003	= .971	− .315	< .001	.164	< .001
CVLT short delay free recall	− .068	= .333	− .338	< .001	.169	< .001
CVLT short delay cued recall	− .033	= .645	− .300	< .001	.134	< .001
CVLT long delay free recall	− .098	= .161	− .299	< .001	.168	< .001
CVLT long delay cued recall	− .097	= .164	− .303	< .001	.171	< .001
Motor dexterity						
Grooved Pegboard time to place with preferred hand	− .125	= .085	.292	< .001	.110	< .001
Grooved Pegboard time to remove with preferred hand	.312	< .001	.174	= .014	.102	< .001
Grooved Pegboard time to place with non-preferred hand	− .098	= .157	.383	< .001	.180	< .001
Grooved Pegboard time to remove with non-preferred hand	.397	< .001	.218	= .002	.165	< .001

Adjusted *R*^2^ values are for the whole model fit

There were also many unique effects of age. Older age predicted lower performance in Corsi Block Tapping, slower CTT1 and CTT2 completion, lower scores in RFFT and WCST, lower recall in CVLT, and slower placing pegs with preferred and non-preferred hand in GPT (all effects *p* < .002). No unique effects of HIV infection were found. Analyses designed to elucidate HIV-age interaction effects on neuropsychological outcomes have not demonstrated significant results.

### Effects of HIV and age on brain volume

The effects of HIV status and aging on brain regional volume were considered, including the results of HIV+ and HIV− subgroups taking part in brain imaging assessment. After the Bonferroni correction, the *p* value was set to be *p* < 0.05/total number of variables tested, i.e., 2 predictors (HIV, age) + 1 covariate (Vocabulary) + 44 brain regional volumes = 0.002 for each predictor effect. Regression coefficients shown in Table 3 demonstrate that being seropositive was not associated with lower brain regional volumes as measured by brain morphometry in comparison to control subjects. Unique significant effects of age revealed that in both HIV+ and HIV− groups, older age was associated with lower cortical gray matter volume and lower total gray matter volume, as well as bilaterally lower volume in putamen and nucleus accumbens (all *p* < .001). Older age was also associated with greater volume of the choroid plexus bilaterally, right lateral ventricle, third ventricle, and white matter hypointensities (all *p* < .001). Although not significant, effects of HIV and age were also found in the variability of cortical white matter volumes, showing HIV-related decreases (all *p* < .02). Finally, the analyses investigating the moderating effects of age on the relationship between HIV status and on regional brain volumes revealed no significant interactions.

**Table 3:** Significant effects of HIV and age on brain regional volumes in HIV+ and HIV− MRI subsamples

Brain region	HIV		Age			
	*β*	*p*	*β*	*p*	Adjusted *R*^2^	*p*
Cortical white matter						
Total	− .248	< .01	.247	< .01	.105	< .002
Left hemisphere	− .248	< .01	.239	< .01	.108	< .002
Right hemisphere	− .221	< .02	.251	< .01	.100	< .005
Cortical gray matter						
Total	.027	= .757	− .442	< .001	.177	< .001
Left hemisphere	.002	= .982	− .448	< .001	.179	< .001
Right hemisphere	.052	= .553	− .430	< .001	.172	< .001
Total gray matter	.014	= .876	− .437	< .001	.167	< .001
Subcortical						
Left putamen	− .095	= .288	− .402	< .001	.149	< .001
Right putamen	− .071	= .423	− .444	< .001	.171	< .001
Left accumbens	− .101	= .264	− .388	< .001	.141	< .001
Right accumbens	− .140	= .103	− .495	< .001	.230	< .001
Left choroid plexus	.148	= .106	.363	< .001	.120	< .001
Right choroid plexus	.190	< .05	.403	< .001	.158	< .001
Right lateral ventricle	.122	= .149	.510	< .001	.251	< .001
Third ventricle	.123	= .139	.547	< .001	.275	< .001
White matter hypointensities	.009	= .924	.415	< .001	.149	< .001

Volumetric measures have been adjusted by total intracranial volume. Adjusted *R*^2^ values are for the whole model fit

### Effects of HIV-associated clinical factors and age on neuropsychological performance and brain volume

The effects of clinical factors, i.e., nadir CD4 cell count, CD4 cell count during participation in the study, duration of HIV infection, duration of cART, and age on neuropsychological performance, were estimated, including the data of all HIV+ participants (*n* = 91). After correcting for multiple comparisons, *p* value was set to be *p* < 0.05/number of predictors (age and 4 clinical parameters) + 1 covariate (Vocabulary) + 26 dependent variables = .0016 for each predictor effect. The effects of clinical factors and age on brain volume were investigated, including the data of HIV+ participants that underwent MRI assessment (*n* = 54). Here, significance level was set to be *p* < 0.05/5 predictors (age and 4 clinical parameters) + 1 covariate (Vocabulary) + 44 brain regional volumes = .001 for each predictor effect. Such levels of significance were also applied in analyses designed to explore interactions.

The studied clinical parameters yielded no significant independent effects on cognitive performance or brain regional volumes. When addressing the interactions, a moderating effect of age on the relationship between duration of cART and time needed to place with non-preferred hand in GPT was observed (*F*(1,78) = 15.070, *p* < .0002, *R*^2^-change = .11, *b =* .006, *t*(78) = 3.88 *p* < .0005). Duration of cART influenced GPT performance in HIV+ participants aged over 53.1 years (*b* = .101, *t*(78) = 3.463, *p* < .001), in which every year on cART increased time of performance in GPT (Plots 1 and 2).

**Plot 1:**
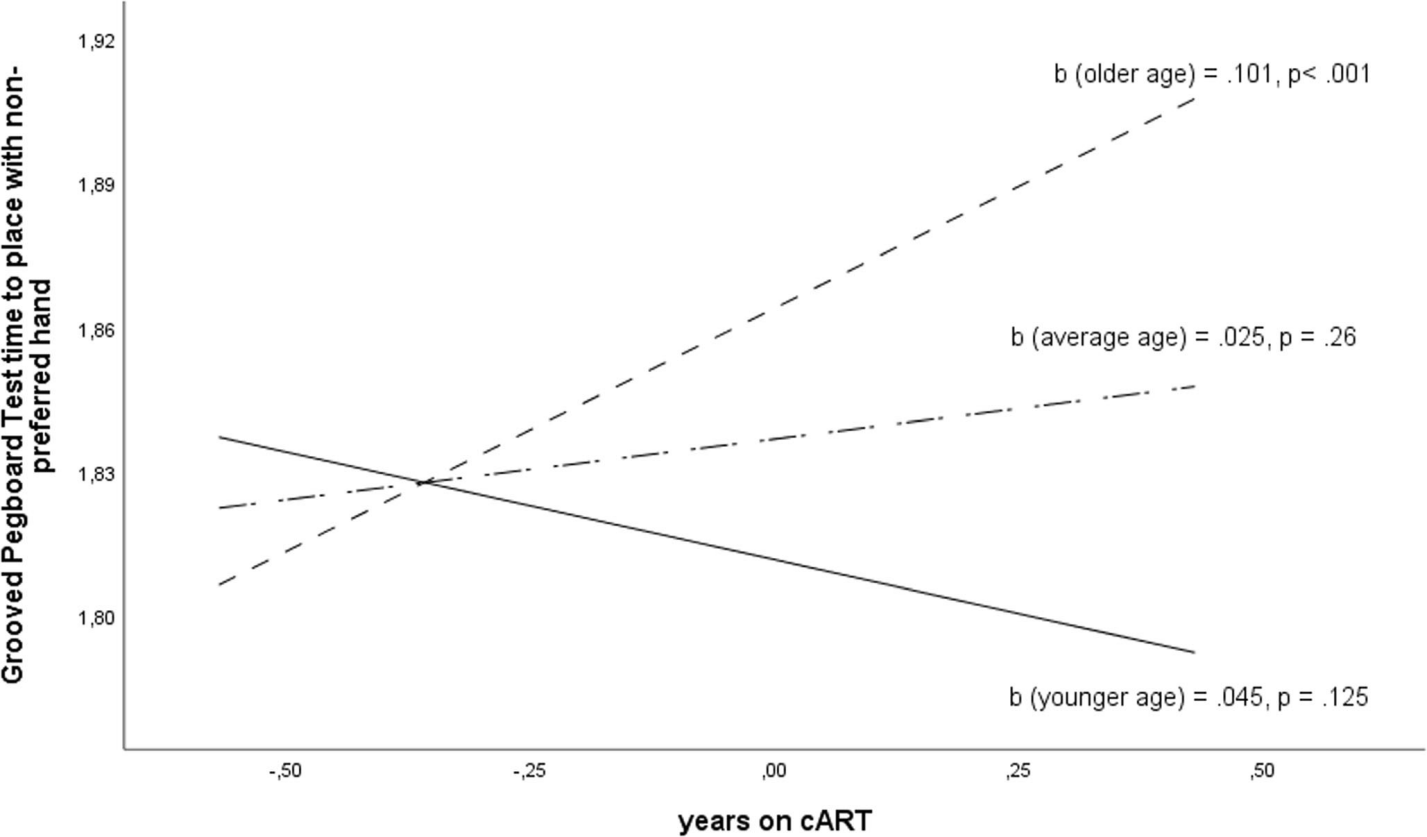
The moderating effect of age on the relationship between the duration of cART and the time to place pegs with non-preferred hand in Grooved Pegboard Test in groups of HIV+ participants of mean age 29.3 years (younger), 41.2 (average), 53.1 (older). Variables are mean centered

**Plot 2:**
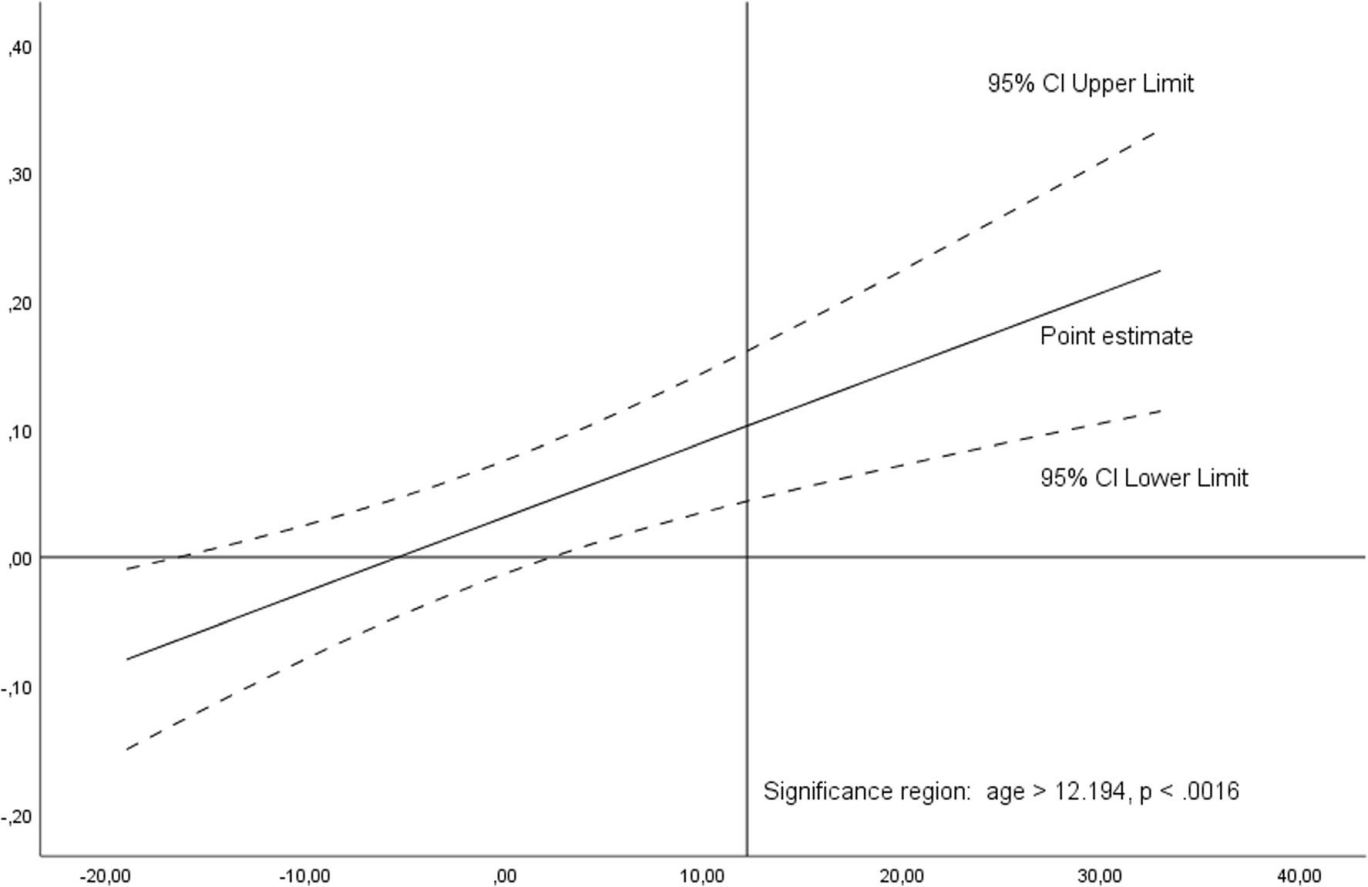
Significance region of the moderating effect of age on the relationship between the duration of cART and the time to place pegs with non-dominant hand in Grooved Pegboard Test based on Johnson-Neyman output. Variables are mean centered. Significance region is situated for age > 12.1 years from the mean, *p* < .0016, i.e., > 53.4 years

Age also moderated the relationship between duration of cART and time needed to remove pegs with non-preferred hand in GPT (*F*(1,78) = 10.164, *p* = .002, *R*^2^-change = .11, *b =* .005, *t*(78) = 3.18 *p* = .002, near to the expected *p* = .0016). The degree to which duration of cART influenced GPT performance was significant in HIV+ participants aged over 53.1 years (*b* = .907, *t*(78) = 2.871, *p* = .005), in which every year on cART increased GPT time of performance (Plots 3 and 4).

**Plot 3:**
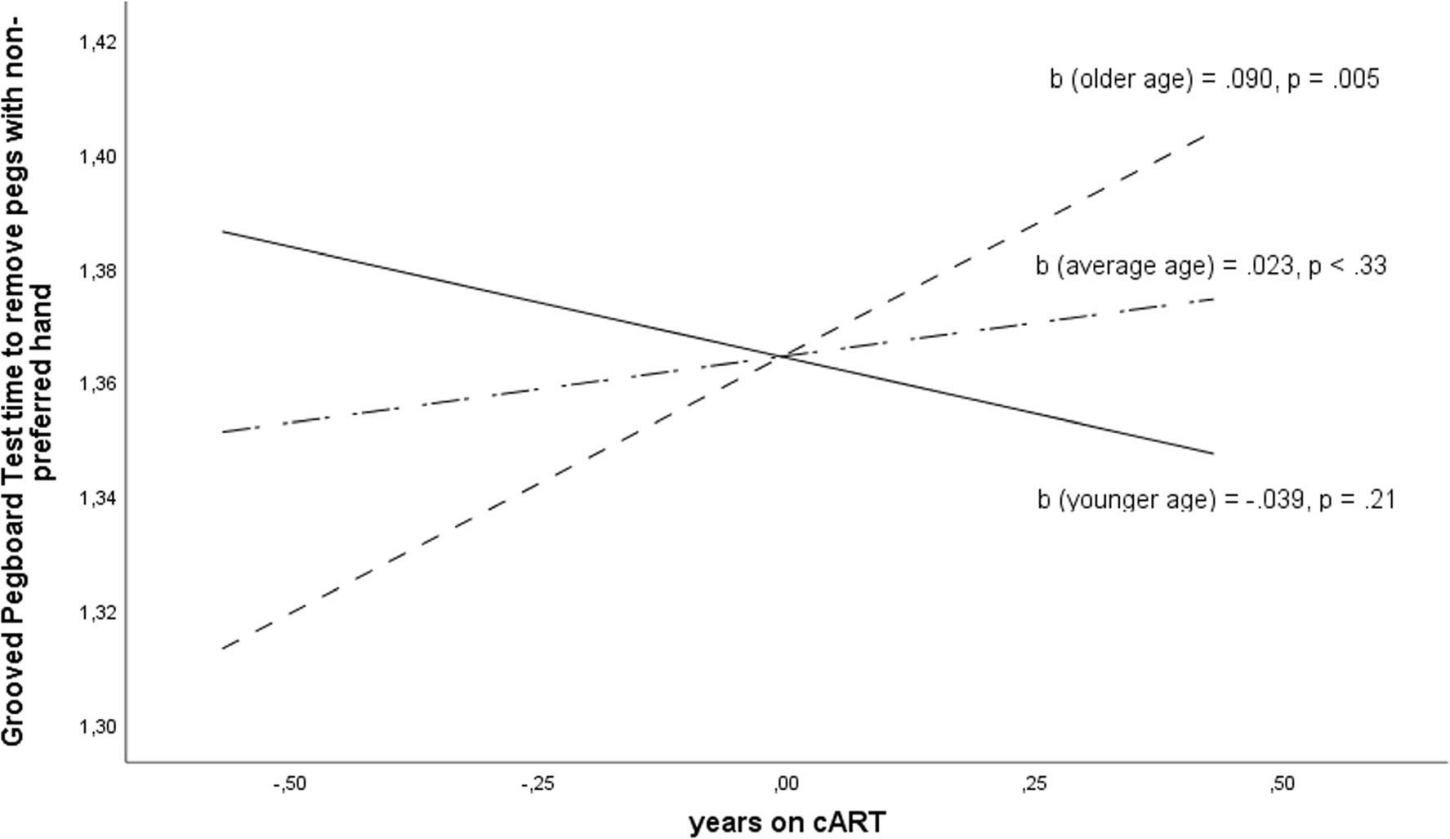
The moderating effect of age on the relationship between the duration of cART and the time to remove pegs with non-dominant hand in Grooved Pegboard Test in groups of HIV+ participants of mean age 29.3 years (younger), 41.2 (average), 53.1 (older). Variables are mean centered

**Plot 4:**
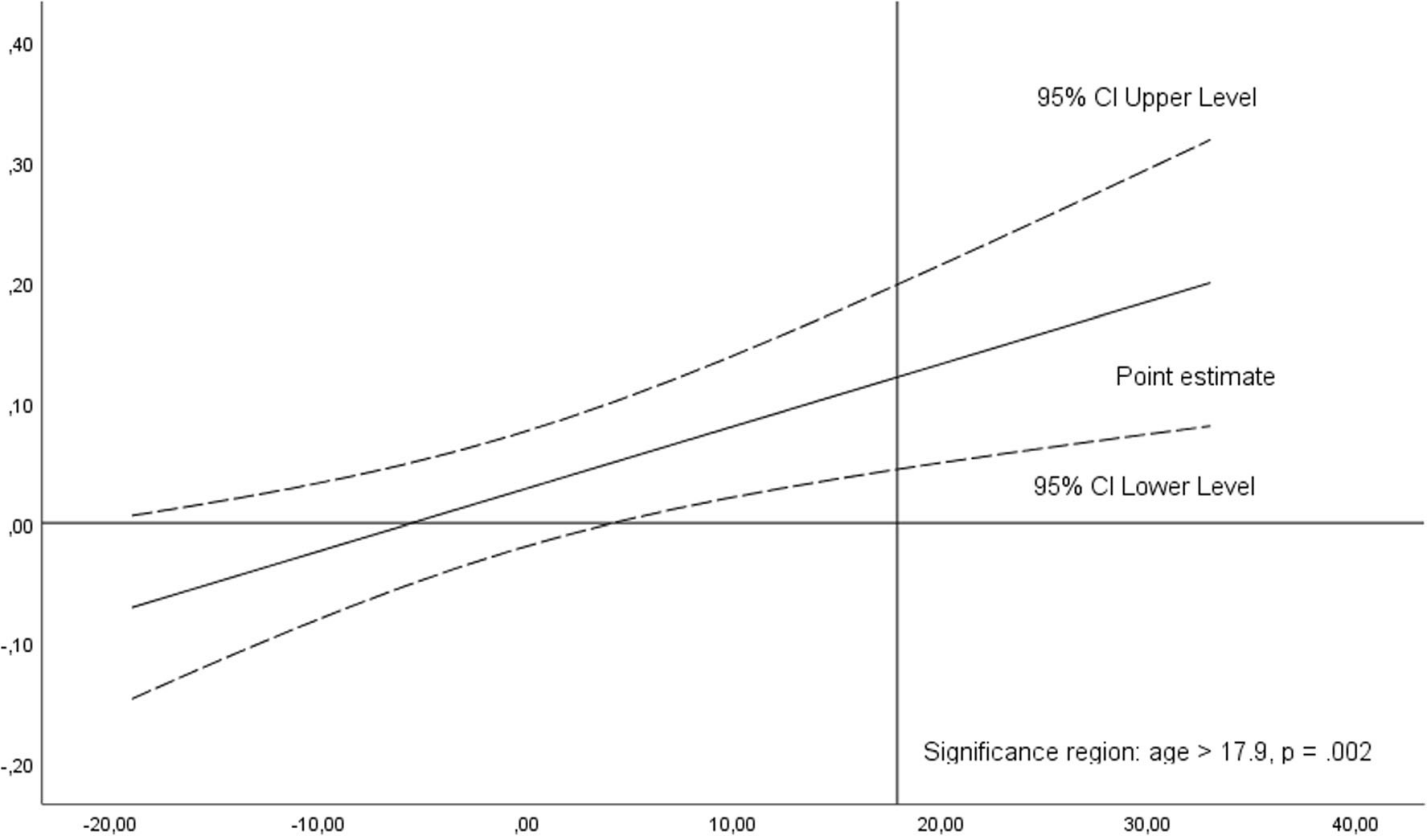
Significance region of the moderating effect of age on the relationship between the duration of cART and the time to remove the pegs with non-dominant hand in Grooved Pegboard Test based on Johnson-Neyman output. Variables are mean centered. Significance region is situated for age > 17. 9 years from the mean, *p* = .002, i.e., > 59.1 years

## Discussion

The results of this study show that for well-educated HIV+ men of various ages, with viral suppression and no active comorbidities, (1) HIV and age led to neuropsychological declines; (2) older age but not HIV was associated with less volume in several cortical and subcortical brain regions; (3) essentially, the adverse effects of HIV infection and age were independent; and (4) in the oldest HIV+ participants, older age decreased speed of visuomotor performance along with every year on cART.

Overall, such findings are consistent with previous evidence showing that HIV infection is associated with cognitive declines despite successful and, in some cases, long-term viral suppression due to cART (Coban et al. 2017; Crum Cianflone et al. 2013; Seider et al. 2014; Sheppard et al. 2015; Simioni et al. 2010). Our results also support prior observations that HIV and age exert independent but not interactive effects on cognitive performance (Ances et al. 2012; Cysique et al. 2011; Valcour et al. 2011). Such results are relevant to the debate on the relationship between HIV and aging because they seem to contradict the hypothesis that age is an additive risk for neuropsychological dysfunction among HIV+ individuals. On the other hand, according to the literature, it is possible that greater cognitive declines among older HIV+ adults compared to demographically similar controls would emerge in follow-up assessments rather than cross-sectionally (Saktor et al. 2010; Seider et al. 2014).

Another important finding is that we have not found volume decreases of gray matter related to HIV infection and the observed HIV-associated loss of cortical white matter as well as the enlargement of the fourth ventricle was not significant after correcting for multiple comparisons. Such results are in line with evidence suggesting that in virally suppressed patients, brain destruction due to HIV replication and inflammation may develop slowly and subtly, starting in subcortical regions (Ances et al. 2012; Corrêa et al. 2016; Cysique et al. 2017; Gelman 2015). The impact of HIV and aging on individual cortical regions in our sample was subject of a separate study conducted with machine-learning technique that has revealed that the best between-group classification accuracy was obtained based on volumetric measures of subcortical regions such as white matter; 3rd, 4th, and lateral ventricles; amygdala; caudate; and putamen (Pluta et al. in press).

This study has also failed to demonstrate eminent HIV-age interactive effects on brain volume, suggesting that advanced age does not accelerate changes associated with HIV. This finding matches the results of a similar cross-sectional study addressing HIV and age effects on the brain in younger and older aviremic patients (Towgood et al. 2012), albeit in that sample, the HIV+ group had less gray matter in several regions when compared to HIV− controls. Interestingly, the present study has proven interactive effects of age and duration of cART, showing that in older HIV+ patients, longer exposure to cART increased the risk of motor slowing. Such effects were limited to performance in GPT and should be confirmed in other motor tasks for more solid conclusions. The possible neuroanatomical changes underlying motor decline in HIV+ subjects may be disrupted cortico-striatal networks and decrease of functional connectivity (Ortega et al. 2015). Our results indicate detrimental effects of cART in the oldest HIV+ participants. However, declines in fine motor performance do not necessarily have to be related only to cART, which in our sample was administered in standard schedules (2 NRTI + PI/r 55.7% of all HIV+ participants, 2NRTI + NNRTI 22.7%, other 20.6%). Literature has emphasized that the etiology of cognitive decline or brain abnormalities in older patients with HIV is multifactorial. In addition to side effects of cART, the involved processes may include chronic inflammation, neurovascular abnormalities, and metabolic and age-related changes (Clark Cohen 2010; Gelman 2015). The literature on the outcomes of cART has been inconclusive. Many prior studies that have examined aviremic individuals with normal levels of CD4 counts reported negative influence of long-term cART on cognitive status or brain (Ellis et al. 2011; Muñoz-Moreno et al. 2008; van den Dries et al. 2017; Walker Brown 2017), while others have found beneficial effects such as higher brain functional connections (Ortega et al. 2015). Future research on the effects of cART in HIV+ population should comprise larger groups of older individuals.

Despite that, on the whole, our results seem to reflect the efficacy of cART in preventing neuroinflammation due to HIV and subsequent extensive neural and glial dropout (Gelman 2015; Hong Banks 2015) as well as notable cognitive impairment. Such belief is supported by the fact that in our sample most patients had cART introduced immediately after HIV diagnosis (51.6%) or within 1 year (19%) or 2 years (11%), and all were successfully suppressed at the moment of the assessment. Although promising, our results should be treated with caution. The sample was relatively young (mean age in HIV+ group 41.2 ± 11.9 vs HIV− group 44.3 ± 12.5; 19 HIV+ subjects age > 50 years vs. 35 controls) and free of active opportunistic infections or comorbidities that may elicit structural brain injury such as cardiovascular conditions or diabetes.

The lack of association between other studied clinical variables (e.g., nadir CD4 cell count, current cell count, duration of HIV infection) and cognition or brain volume also may reflect the small degree of infection severity in the studied sample (28.6% HIV+ participants experienced nadir CD4 > 200 cells/μL) and/or the successful cessation of brain injury due to quick suppression. This result differs from prior evidence revealing low nadir CD4 as an important risk factor for cognitive changes (Ellis et al. 2011) and volume decreases in the putamen (Wright et al. 2016), thalamus (Cohen et al. 2010), white matter (Hua et al. 2013), and other subcortical regions (Jerningan et al. 2011; Sanford et al. 2017). Still, the role of T cells in the development of brain abnormalities and cognitive deficits in HIV patients is not completely understood. Contradictory hypotheses have attributed CD4 cells a protective role against neuroinflammation or a contributory role to it in virally suppressed patients. Other factors such as aging or past comorbid conditions also may have an effect (Hong Banks 2015).

The differences between our results and other studies may be due to dissimilarities in demographic, clinical, treatment, or other characteristics. To minimize confounding factors, we enrolled only men with secondary or university education and who were employed, without substance abuse, psychiatric diseases, or dementia. HIV+ participants were homogeneously neurologically asymptomatic, aviremic, and without HCV co-infection or opportunistic or sexually transmitted diseases at the time (although a considerable proportion of the HIV+ group had been diagnosed with syphilis, neurosyphilis, or HCV in the past). Most of the HIV+ participants were cognitively normal according to HAND criteria (81.5% were classified as no HAND, Egbert et al. 2018). This differentiates our sample from other recent research that pooled participants of both sexes, multiple races/ethnicities, patients with different levels of viremia, HCV co-infection, neurocognitive impairment, depression, heterogeneous socioeconomic background, substance use disorders, etc. (e.g., Coban et al. 2017; Cohen et al. 2010; Sanford et al. 2017; Sheppard et al. 2015).

Such characteristics of our HIV+ group may reflect the existent clinical observations of patients undergoing treatment and provide important information about the Polish male HIV+ sample on cART. They constrain, however, generalization of our findings to healthy, educated, wealthy, and demographically/clinically homogeneous cohorts (e.g., Crum Cianflone et al. 2013; Cysique et al. 2011; McDonnell et al. 2014; Seider et al. 2014; Simioni et al. 2010). Further studies on larger samples are needed to determine if brain reductions occur in such samples despite successful implementation of cART. If CD4 drop mechanisms or duration of exposure to cART contribute to such a process remains an open question.

This study has some limitations. First, it was cross-sectional, and we collected the data only once. Follow-up assessments are necessary to directly estimate if cognition or brain volume differentially decline as a function of HIV status and age in aviremic HIV+ cohorts. This is particularly important in older patients who get the infection at older ages and are supposed to be more vulnerable. Another limitation is that we did not analyze the impact of past comorbid conditions on cognition and brain structure—conditions such as AIDS-associated opportunistic infections and sexually transmitted diseases (STDs) such as syphilis, neurosyphilis, gonorrhea, and chlamydia. Comorbid conditions may increase the risk of neurocognitive decline in HIV+ patients (Heaton et al. 2015; van den Dries et al. 2017). Other co-infections, such as HCV, also can lead to brain inflammation and injury, which might exacerbate further the aging process in the HIV-infected brain (Holt et al. 2012). Less is known about AIDS-associated opportunistic infections, including the herpes virus, cytomegalovirus, and STDs such as syphilis. These diseases can lead to severe neurological complications but few studies have evaluated their impact on the HIV-infected brain, especially the aging brain (Hong Banks 2015). The relationships among history of comorbid conditions, advanced age, and cognitive or brain declines in successfully treated HIV+ adults remain uncertain.

Despite the limitations, the findings of this study provide a unique contribution to the existing literature on aging with HIV infection and have important potential clinical implications for neuropsychological outcomes in contemporary HIV+ cohorts where a substantial number of people are older than 50. The results underscore the importance of monitoring neuropsychological functioning and brain structure in chronically infected patients. This is extremely important in older patients with long adherence to cART therapies who may be at greatest risk of developing cognitive and brain abnormalities. The results of the present study also support recommendations that early antiretroviral intervention may offer an effective approach to prevent or lessen brain atrophy and cognitive decline over time, although further research is needed to confirm this.

## Electronic supplementary material

Appendix Table 1 (DOCX 16 kb)
