# Supplementary material for: Effects of age, HIV, and HIV-associated clinical factors on neuropsychological functioning and brain regional volume in HIV+ patients on effective treatment
Source: J Neurovirol. 2018 Oct 8;25(1):9–21. doi: 10.1007/s13365-018-0679-4 (PMC6416454; doi:10.1007/s13365-018-0679-4)
Supplement: Supplementary file 1 — (DOCX 16 kb) [file 13365_2018_679_MOESM1_ESM.docx]

**Appendix**

Table 1. Neuropsychological characteristics

| Test variable | HIV+  N=91  M (SD) | HIV-  N=95  M (SD) | *t* | Group differences | MRI HIV+  N=54  M (SD) | MRI HIV-  N=62  M (SD) | *t* | Group differences |
| --- | --- | --- | --- | --- | --- | --- | --- | --- |
| **Attention / Working memory** |  |  |  |  |  |  |  |  |
| Corsi Block Tapping forward | 8.3 (1.9) | 9 (1.8) | <.005 | HIV- > HIV+ | 8.2 (1.8) | 9.3 (1.9) | <.005 | HIV- > HIV+ |
| Corsi Block Tapping backward | 7.6 (1.8) | 8.8 (1.8) | <.001 | HIV- > HIV+ | 7.6 (1.5) | 9 (1.7) | <.001 | HIV- > HIV+ |
| WAIS–R (PL) Digit Span forward | 6 (1.9) | 6.7 (1.8) | <.05 | HIV- > HIV+ | 6.3 (1.9) | 6.8 (1.8) | NS |  |
| WAIS–R (PL) Digit Span backward | 5.6 (1.8) | 7 (2.6) | <.001 | HIV- > HIV+ | 5.6 (1.8) | 6.8 (2.7) | <.01 | HIV- > HIV+ |
| CTT1 time | 44 (18.8) | 42.2 (16.4) | NS |  | 42.8 (12.8) | 43.1 (16.7) | NS |  |
| CTT2 time | 88.1 (38) | 83.1 (28.8) | NS |  | 86.4 (32.6) | 85 (31.2) | NS |  |
| **Executive** |  |  |  |  |  |  |  |  |
| WCST total correct responses | 73.6 (8.7) | 70 (10.1) | <.05 | HIV+ > HIV- | 72.1 (8.4) | 70 (9.5) | NS |  |
| WCST percent errors | 23.4 (10.9) | 22.2 (13.3) | NS |  | 21.9 (10.7) | 21.1 (12.4) | NS |  |
| WCST percent perseverative responses | 13.6 (7.3) | 13.6 (11.4) | NS |  | 12.7 (7) | 12.8 (9.3) | NS |  |
| WCST percent conceptual responses | 67.5 (17) | 69.7 (17.7) | NS |  | 69.4 (17.6) | 70.9 (16.4) | NS |  |
| WCST categories completed | 5.3 (1.3) | 5.3 (1.4) | NS |  | 5.6 (1.1) | 5.5 (1.3) | NS |  |
| RFFT unique designs | 84.8 (26.3) | 98.9 (24.7) | <.001 | HIV- > HIV+ | 83.4 (25.4) | 101.2 (25.1) | <.001 | HIV- > HIV+ |
| RFFT perseverations | 8.4 (8.8) | 8.1 (8.1) | NS |  |  |  |  |  |
| **Motor dexterity** |  |  |  |  |  |  |  |  |
| Grooved Pegboard time to place with preferred hand | 65.5 (13.8) | 67.8 (11.5) | NS |  | 64.1 (10.6) | 68.2 (12) | NS |  |
| Grooved Pegboard time to remove with preferred hand | 23.5 (4.2) | 21.2 (3.3) | <.002 | HIV+ > HIV- | 23.7 (3.6) | 21.6 (3.5) | <.005 | HIV+ > HIV- |
| Grooved Pegboard time to place with non-preferred hand | 72.9 (14.9) | 75.3 (13.5) | NS |  | 71.1 (12.8) | 75.3 (13.9) | NS |  |
| Grooved Pegboard time to remove with non-preferred hand | 24.2 (4.2) | 22.1 (9.1) | <.001 | HIV+ > HIV - | 24.2 (3.8) | 21 (3.6) | <.001 | HIV+ > HIV- |
| **Learning** |  |  |  |  |  |  |  |  |
| CVLT list A trials 1-5 | 55.5 (10.2) | 54.8 (8.7) | NS |  | 56 (9.6) | 55.9 (8.7) | NS |  |
| CVLT list B | 6.6 (1.9) | 6.7 (1.8) | NS |  | 6.7 (1.8) | 6.8 (1.9) | NS |  |
| CVLT short-delay free recall | 11.4 (3.1) | 11.9 (2.5) | NS |  | 11.5 (2.8) | 12.2 (2.4) | NS |  |
| CVLT short-delay cued recall | 12.3 (2.2) | 12.6 (2) | NS |  | 12.3 (2) | 12.6 (1.9) | NS |  |
| CVLT long-delay free recall | 11.9 (2.8) | 12.5 (2.3) | NS |  | 11.9 (2.5) | 12.5 (2.2) | NS |  |
| CVLT long-delay cued recall | 12.4 (2.5) | 12.8 (2) | NS |  | 12.4 (2.2) | 12.8 (1.9) | NS |  |
| CVLT percent recall primacy region | 26.6 (5.3) | 28.2 (4.7) | <.05 | HIV+ > HIV - |  |  |  |  |
| **Language** |  |  |  |  |  |  |  |  |
| WAIS–R (PL) Vocabulary | 41.8 (9.3) | 46.3 (8.4) | <.002 | HIV- > HIV+ | 42.4 (9.2) | 46.5 (8) | .05 | HIV- > HIV+ |
| Verbal Fluency | 113.9 (25.4) | 119.5 (24.9) | NS |  | 116.9 (26.2) | 121.6 (22.4) | NS |  |

Note: NS = not significant; *SD* = standard deviation.
